# Structures and Functions of Qβ Replicase: Translation Factors beyond Protein Synthesis

**DOI:** 10.3390/ijms150915552

**Published:** 2014-09-02

**Authors:** Kozo Tomita

**Affiliations:** National Institute of Advanced Industrial Sciences and Technology (AIST), Biomedical Research Institute, 1-1-1 Higashi, Tsukuba, Ibaraki 305-8566, Japan; E-Mail: kozo-tomita@aist.go.jp; Tel./Fax: +81-298-61-6085

**Keywords:** Qβ replicase, RNA-dependent RNA polymerase, EF-Tu, EF-Ts, ribosomal protein S1

## Abstract

Qβ replicase is a unique RNA polymerase complex, comprising Qβ virus-encoded RNA-dependent RNA polymerase (the catalytic β-subunit) and three host-derived factors: translational elongation factor (EF) -Tu, EF-Ts and ribosomal protein S1. For almost fifty years, since the isolation of Qβ replicase, there have been several unsolved, important questions about the mechanism of RNA polymerization by Qβ replicase. Especially, the detailed functions of the host factors, EF-Tu, EF-Ts, and S1, in Qβ replicase, which are all essential in the *Escherichia coli* (*E. coli*) host for protein synthesis, had remained enigmatic, due to the absence of structural information about Qβ replicase. In the last five years, the crystal structures of the core Qβ replicase, consisting of the β-subunit, EF-Tu and Ts, and those of the core Qβ replicase representing RNA polymerization, have been reported. Recently, the structure of Qβ replicase comprising the β-subunit, EF-Tu, EF-Ts and the *N*-terminal half of S1, which is capable of initiating Qβ RNA replication, has also been reported. In this review, based on the structures of Qβ replicase, we describe our current understanding of the alternative functions of the host translational elongation factors and ribosomal protein S1 in Qβ replicase as replication factors, beyond their established functions in protein synthesis.

## 1. Introduction

Qβ virus has a single positive strand RNA genome. It infects *Escherichia coli* (*E. coli*) and replicates by transcribing its genomic RNA with Qβ replicase [1]. Qβ replicase was initially isolated by Haruna and Spiegelman almost fifty years ago [2]. Since then, Qβ replicase has fascinated researchers in the field of RNA enzymology because of its unique composition, and several interesting and important, yet unsolved, problems remain for Qβ replicase [3].

**Figure 1:**
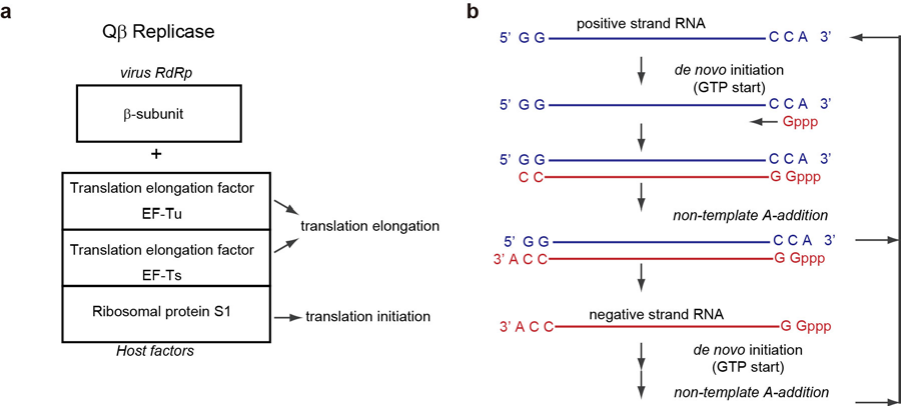
Composition of Qβ replicase and replication cycle of Qβ RNA. (**a**) Composition of Qβ replicase. Qβ replicase consists of the virus-encoded RNA-dependent RNA polymerase (β-subunit), and three host translation factors: elongation factor (EF)-Tu, EF-Ts, and ribosomal protein S1; (**b**) Replication cycle of Qβ RNA. Qβ virus has a single positive strand RNA. The positive and negative strand Qβ RNAs both have 5'-GG and CCA-3' sequences. A-3' does not serve as a template nucleoside, and is added at the terminal stage of RNA synthesis without a nucleic acid template.

Qβ replicase is a tetrameric protein complex of the virus-encoded RNA-dependent RNA polymerase (RdRp, β-subunit) and three host-derived factors (Figure 1a). The β-subunit is the catalytic subunit for RNA-dependent RNA polymerization, and the host factors include the translational elongation factor (EF)-Tu, EF-Ts and ribosomal protein S1 [1,4,5,6,7]. The core complex, consisting of the β-subunit, EF-Tu and EF-Ts, has RNA polymerization activity *in vitro*, and the assembly of the β-subunit with EF-Tu and EF-Ts is reportedly required for the RNA polymerization activity. S1 is necessary for the efficient replication of Qβ RNA in the host *E. coli*, and interestingly, it is dispensable for the positive strand RNA synthesis from the negative strand RNA [1,6,8,9,10]. EF-Tu, EF-Ts, and S1 are all essential protein synthesis factors in the host *E. coli*.

The established role of EF-Tu in protein synthesis is to deliver an aminoacyl-tRNA in its GTP-bound form to the ribosome [11]. After a codon-anticodon match is made on the ribosome, GTP is hydrolyzed to GDP, and the GDP-bound form of EF-Tu is released from the ribosome [12,13]. EF-Ts binds GDP-bound EF-Tu, displaces the GDP, and recycles EF-Tu for the next round of the elongation cycle of protein synthesis. Ribosomal protein S1 is the largest protein of the 30S ribosome, and is essential for the translation of most mRNAs [14]. In particular, S1 is required for the efficient translation initiation of most natural mRNAs containing a weak or lacking a Shine-Dalgarno (SD) sequence, as well as mRNAs containing structured 5'-leader sequences [15,16,17,18,19,20]. The detailed molecular mechanism of RNA polymerization by Qβ replicase containing these three host factors, and their functions in the replication and transcription of Qβ RNA had remained elusive.

The positive and negative strand RNAs of Qβ virus both have CCA-3' and 5'-GG sequences [21,22] (Figure 1b). The 3'-terminal A (3'-A) does not serve as a template nucleoside; instead, the 3'-penultimate C functions as the first template nucleoside. Thus, the RNA polymerization by Qβ replicase starts with GTP, without using an RNA primer (*de novo* initiation) [1,23,24]. The 3'-A is added by the intrinsic terminal nucleotidyltransferase activity of Qβ replicase at the final stage of RNA replication [1,25,26]. The mechanism of *de novo* initiation of RNA polymerization by Qβ replicase had remained enigmatic. In addition, the mechanisms of RNA polymerization termination and non-template 3'-A-addition by Qβ replicase, and the functions of the 3'-A in both the positive and negative strand Qβ RNAs were also unknown.

During the last five years, the crystal structures of the core Qβ replicase (β-subunit, EF-Tu and EF-Ts) and its complexes with various RNAs (template and growing RNAs) representing RNA polymerization have been reported [27,28,29,30]. Recently, the crystal structure of Qβ replicase containing the *N*-terminal half of S1, which is capable of the initiation of Qβ RNA replication, was solved [31]. These crystallographic studies have answered several, but not all, of the long-standing questions about the unique Qβ replicase.

In the present review, we describe our current understanding of the extra-translation functions of the host factors of Qβ replicase, and the mechanisms of initiation and termination of RNA polymerization by Qβ replicase, based on the crystallographic structural studies.

## 2. Overall Structure of the Core Qβ Replicase

### 2.1. Interactions between Subunits

**Figure 2:**
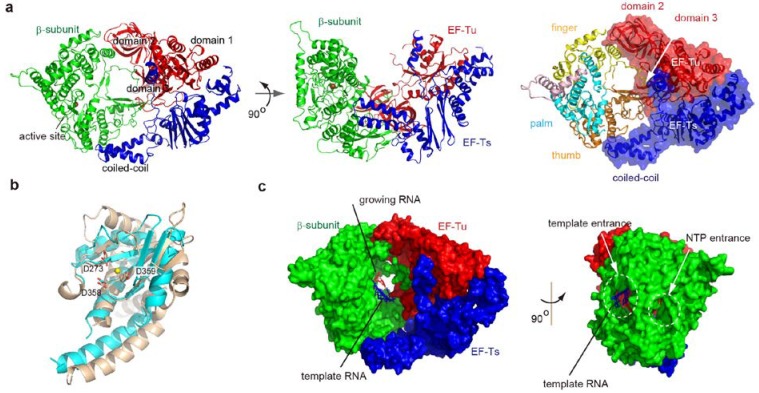
Structure of core Qβ replicase. (**a**) Overall structure of the core Qβ replicase. The β-subunit, EF-Tu and EF-Ts are colored green, red and blue, respectively (**left**). Interactions between subunits (**right**). The β-subunit consists of three domains, finger (yellow), palm (cyan) and thumb (orange); (**b**) Structures of the catalytic palm domains of the β-subunit (cyan) and phi-6 RdRp (brown). Catalytic carboxylates are depicted by stick models; (**c**) A model of RNA synthesis identified possible substrate tunnels in the core Qβ replicase. The double stranded RNA is shown, in which the template and growing RNAs are depicted by blue and red stick models, respectively.

Five years ago, the crystal structures of the core Qβ replicase, consisting of the β-subunit, EF-Tu and EF-Ts, were reported by two independent groups [27,28]. The overall structure of the core Qβ replicase resembles a boat, and the three subunits are tightly assembled. The stoichiometry of the β-subunit, EF-Tu, and EF-Ts in the asymmetric unit is 1:1:1 (Figure 2a).

The β-subunit adopts a typical right-handed structure consisting of three domains, finger, palm and thumb, as in other RdRps [32,33]. The catalytic residues reside in the palm domain. The EF-Tu and EF-Ts subunits in Qβ replicase interact tightly, as in the EF-Tu:EF-Ts binary complex [34], and slight differences between the structures are observed in the regions interacting with the β-subunit. Domains 2 and 3 of EF-Tu are the recognition domains of the acceptor and the TψC stem and loop of aminoacyl-tRNA, together with domain 1 [35,36]. The finger domain of the β-subunit interacts with domain 2 of EF-Tu, and the thumb domain of the β-subunit interacts with domain 3 of EF-Tu and the coiled-coil domain of EF-Ts. The interactions between the subunits are mainly hydrophobic, and those between the β-subunit and EF-Tu and EF-Ts contribute to the maintenance of the catalytic core structure of the β-subunit. *In vivo* analyses revealed that disruptions of the hydrophobic interactions between the β-subunit and EF-Tu (or EF-Ts) reduced the expression and assembly of the core Qβ replicase [28,37]. Thus, it is likely that EF-Tu and EF-Ts, in the core Qβ replicase, act as chaperones [38,39,40] that assist in the folding of the active β-subunit and the assembly and maintenance of the active core Qβ replicase.

### 2.2. Structure of the β-Subunit

The right-handed structure of the β-subunit is homologous to those of other RdRps [32,33,41]. The catalytic palm domain of the β-subunit consists of five anti-parallel β-sheets flanked by four α-helices, and the structure is homologous to those of other RdRps. The three conserved catalytic carboxylates (Asp273, Asp358, and Asp359) reside in the palm domain, and the geometry of these residues is quite similar to those in other RdRps, such as phi-6 RdRp [32,33,41]. However, only one metal is coordinated by the three carboxylates. Conventional template-dependent DNA/RNA polymerization proceeds by a two-metal catalytic mechanism for the nucleotidyl transfer [42,43]. The two metals are usually coordinated by the three catalytic carboxylates. One metal acts as a general base that activates the 3'-OH group at the 3'-end of the growing RNA, and the other stabilizes the leaving pyrophosphate during the nucleophilic attack by the 3'-OH of the growing RNA. Thus, in the presence of both an incoming nucleotide and an RNA primer, the two metals would be coordinated by the catalytic carboxylates, as described below.

In the catalytic crevasse of the core Qβ replicase, a model of the double stranded RNA, representing the template and growing RNAs, and an incoming nucleotide was built. The model represents the elongation stage of RNA polymerization (Figure 2c). The model identified two possible tunnels in the core Qβ replicase: One for single strand template RNAs and the other for incoming nucleotides. This model also implied that the double stranded RNA between the template and growing RNAs would proceed towards the EF-Tu:EF-Ts in the core Qβ replicase. In addition, the double stranded RNA would be structurally altered or separated, for processive RNA polymerization by Qβ replicase as the RNA chain elongates. This model was evaluated by the structural studies of the core Qβ replicase, representing the initiation and elongation stages of RNA polymerization, as described below.

## 3. Initiation Stage of RNA Polymerization

### 3.1. De Novo Initiation of RNA Synthesis

Both the positive and negative strand Qβ RNAs have CCA-3' [21,22] (Figure 1b). The 3'-terminal A (3'-A) does not serve as a template nucleoside. Instead, the 3'-penultimate C functions as the first template nucleoside, and RNA synthesis starts with GTP without using an RNA primer. Thus, GTP can act as a priming nucleotide [1,23,24]. The 3'-A is reportedly required for the replication of Qβ RNAs by Qβ replicase, and the 3'-terminal nucleoside of these RNAs is predominantly adenosine [44,45]. An *in vitro* biochemical study showed that an RNA either lacking the terminal adenosine or ending with CCU-3' or CCG-3' is less competent as a template for the synthesis of the complementary RNA [29]. This is due to the lower efficiency of the mutant RNAs at the initiation stage of RNA polymerization, rather than at the elongation stage. Thus, the presence of the 3'-A is required for efficient *de novo* initiation. The detailed molecular functions of the 3'-A and the mechanism of *de novo* initiation of RNA synthesis have not been clarified.

### 3.2. Structure of the Initiation Stage

**Figure 3:**
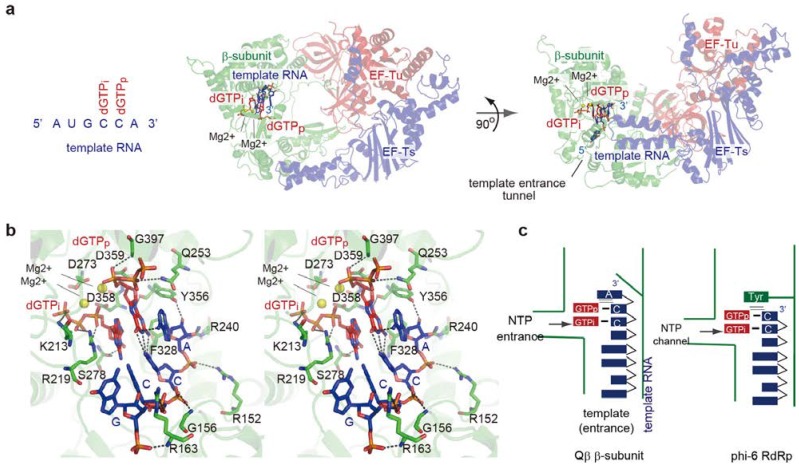
Structure of the *de novo* initiation stage. (**a**) Overall structure of the initiation stage of RNA polymerization. The sequence of a template RNA with CCA-3' is depicted. The β-subunit, EF-Tu and EF-Ts are colored green, red, and blue, respectively; (**b**) Detailed view of the catalytic pocket at the initiation stage. The template RNA and GTP analogs (dGTPp) and (dGTPi) are depicted by stick models; (**c**) Simplified depiction of the structure representing the *de novo* initiation of RNA polymerization by the core Qβ replicase (**left**), and its comparison with the structure representing the *de novo* initiation of RNA polymerization by phi-6 RdRp (**right**). The functions of the 3'-A in Qβ RNA would be equivalent to those of Tyr630 in phi-6 RdRp.

The crystal structure representing the initiation of RNA polymerization was reported recently [28] (Figure 3a). The core Qβ replicase was crystallized in the presence of a short template RNA bearing CCA-3' and a GTP analog (3'-dGTP). In the structure, the CCA- 3' of the template RNA accesses the catalytic site of the β-subunit through the template entrance, and the template RNA only interacts with the β-subunit. Two 3'-dGTPs reside in the catalytic site of the β-subunit, and form Watson-Crick base-pairs with the CC sequence of CCA-3' of the template RNA (Figure 3b). The geometries of the 3'-dGTPs and the two metals (Mg^2+^) relative to the three catalytic carboxylates (Asp273, Asp358 and Asp359) suggested that the structure represents the initiation stage of RNA polymerization, with one 3'-dGTP corresponding to a priming nucleotide (3'-dGTPp) and the other to an incoming nucleotide (3'-dGTPi).

Although the ribose moiety of the 3'-A of the template RNA hydrogen bonds with amino acid residues in the β-subunit, the adenine of 3'-A does not interact with the β-subunit. Instead, the adenine of 3'-A stacks with Watson-Crick hydrogen-bonds between the 3'-dGTPp and the 3'-penultimate C in the template RNA. Watson-Crick hydrogen-bonds are also formed between the 3'-dGTPi and the 3'-second C in the template RNA, which stack with the Watson-Crick hydrogen-bonds between the 3'-dGTPp and the 3'-penultimate C. Thus, the continuous π–π interactions formed at the initiation stage of RNA polymerization stabilize the initiation complex, and the 3'-A of the template RNA provides a stable platform for the construction of the *de novo* initiation complex (Figure 3b,c).

In the phi-6 RdRp, RNA polymerization initiates without using an RNA primer, as in Qβ replicase, but the 3'-terminal nucleoside serves as the first template nucleoside [46,47]. A structural comparison between the Qβ replicase initiation complex and the phi-6 RdRp initiation complex indicated that the position occupied by the 3'-A of the template RNA in the Qβ replicase initiation complex is occupied by the Tyr630 residue in the phi-6 initiation complex [48,49]. The Tyr630 residue in phi-6 RdRp facilitates the efficient initiation of RNA polymerization, without using an RNA primer. Tyr630 stacks on the hydrogen-bonds between the priming nucleotide (GTP) and the 3'-nucleoside (3'-C) of the template RNA, and provides a platform for the construction of the initiation complex (Figure 3c). Thus, in a similar manner to Tyr630 of phi-6 RdRp, the extra nucleoside at the 3'-A of Qβ viral RNA could stabilize the initiation complex for the efficient initiation of Qβ RNA replication. As described below, the 3'-A of Qβ RNA is added, without using a nucleic acid template, at the terminal stage of RNA polymerization (Figure 1b).

## 4. Elongation Stages of RNA Polymerization

### 4.1. Elongation of RNA Synthesis

The model of the RNA elongation stage, in which the double stranded RNA between the template and growing RNAs and an incoming nucleotide are placed in the active crevasse of the core Qβ replicase (Figure 2c), suggested that the double stranded RNA would proceed towards the EF-Tu:EF-Ts in the core Qβ replicase as the RNA chain is elongated [27,28]. The model implied that until around nine to ten nucleotides of RNA are synthesized, the template RNA forms a double strand with the growing RNA, but afterward, as the RNA chain is elongated, the double stranded RNA would be structurally altered or separated into the single stranded RNAs for processive RNA polymerization. Alternatively, it was also suggested that the structures of EF-Tu and EF-Ts in the core Qβ replicase might dynamically change during the RNA elongation stages.

### 4.2. Structure of Elongation Stages

**Figure 4:**
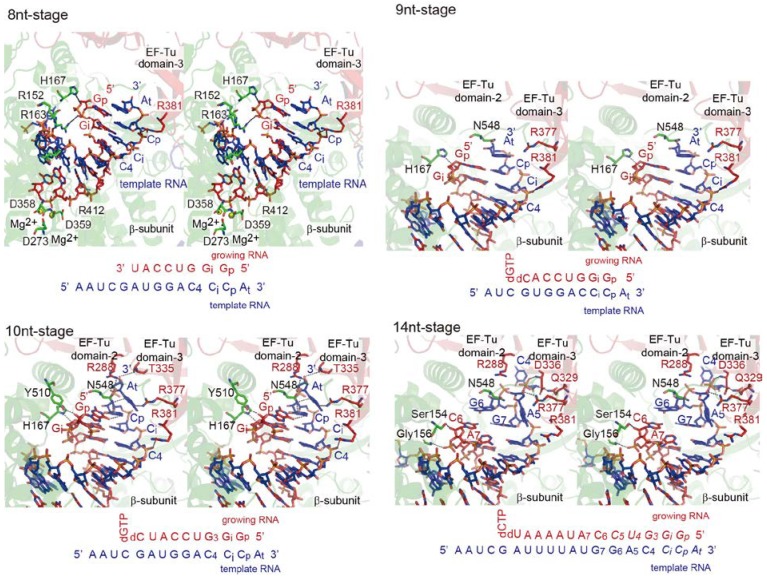
Structures of elongation stages. Structures representing eight, nine, ten and fourteen nucleotide-long synthesized RNAs (8nt-, 9nt-, 10nt- and 14nt-stages). Only the structures in the catalytic crevasse of core Qβ replicase are shown. The β-subunit, EF-Tu and EF-Ts are colored green, red and blue, respectively. The template and growing RNAs are depicted by blue and red stick models, respectively. The sequences of the template RNA and the growing RNA at each stage are depicted.

Several crystal structures of the core Qβ replicase, representing the elongation stages of RNA polymerization, were reported [29] (Figure 4). The core Qβ replicase was crystallized in the presence of a short template RNA bearing CCA-3' and a growing RNA complementary to the template RNA, except for the terminal 3'-A of the template RNA, in either the presence or absence of NTPs or NTP analogs. Pre-incubation of the core Qβ replicase with RNAs, NTPs, and NTP analogs allowed the enzyme to elongate the RNA chain. The structures represent the elongation stages of RNA polymerization, in which seven to ten and fourteen nucleotide-long RNAs were synthesized (hereafter, these stages are referred to as the 7nt-, 8nt-, 9nt-, 10nt- and 14nt-stages).

At the 7nt-stage, the template and growing RNAs form a stable duplex that proceeds from the active site of the β-subunit towards EF-Tu in the core Qβ replicase. As the RNA chain is elongated by one nucleoside (8nt-stage), the duplex between the template and growing RNA is driven towards EF-Tu in the complex. During these primary elongation stages, the phosphate backbone of the 3'-part of the template RNA interacts with domain 3 of EF-Tu and this interaction guides the duplex of the template and growing RNAs toward EF-Tu in the complex.

**Figure 5:**
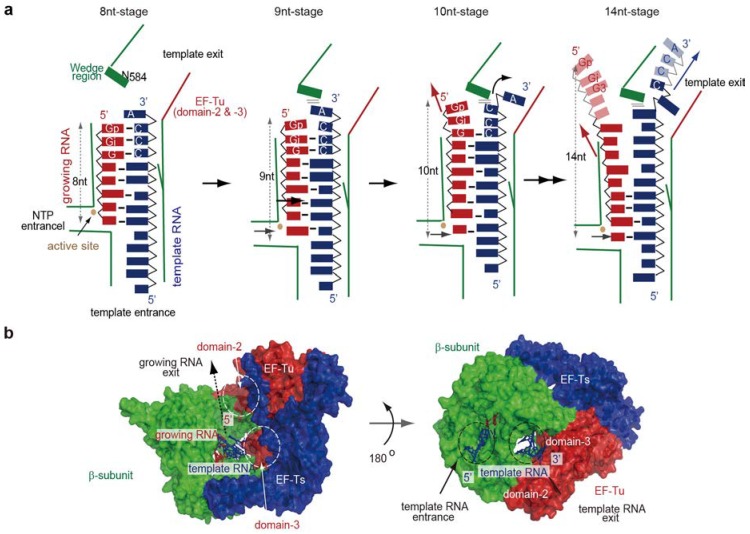
Elongation stages of RNA polymerization. (**a**) Simplified depiction of the structures representing the elongation stages of RNA polymerization by Qβ replicase; (**b**) Template tunnels in the core Qβ replicase. Surface model of the elongation stage, where a fourteen nucleotide-long RNA is synthesized (14nt-stage in Figure 4). The β-subunit, EF-Tu and EF-Ts are colored green, red and blue, respectively. The template and growing RNAs are depicted by blue and red stick models, respectively.

As the RNA chain is elongated by one nucleotide (9nt-stage), the duplex is further driven toward EF-Tu in the complex. At this stage, the 3'-overhanging adenine of the template RNA, in the temporal duplex of the template and growing RNAs, stacks onto the *C*-terminal region of the β-subunit. As a result, the hydrogen-bonds between the template and growing RNAs are compressed, and the duplex is slightly destabilized. Upon elongation of the RNA by one nucleoside (10nt-stage), the temporal duplex is further driven towards EF-Tu in the complex. As a result, the 3'-overhanging adenine in the template RNA flips and enters the internal space between the β-subunit and EF-Tu. The interactions between the 3'-part of the template RNA and domains 2 and 3 of EF-Tu facilitate the relocation of the flipped adenine into the internal space. The hydrogen bonds between the template and growing RNAs are further destabilized, and the *C*-terminal region starts to wedge the duplex apart. At the 14nt-stage of RNA elongation, the 3'-part of the template RNA is completely split apart from the growing RNA, and enters the internal space between the β-subunit and EF-Tu. The template RNA is threaded by the space in the core Qβ replicase. Thus, the internal space is a template exit tunnel (Figure 4 and Figure 5a).

The *C*-terminal region of the β-subunit acts as a wedge that splits the temporal duplex between the template and growing RNAs. This splitting is assisted by the interactions between the template RNA and the basic amino acid residues in EF-Tu. These sequential crystallographic studies suggested that EF-Tu, in the core Qβ replicase, modulates the processive elongation stage of RNA polymerization (Figure 4 and Figure 5a). In the 14nt-stage, the 5'-part of the growing RNA is not clearly visible, suggesting that after it is separated from the template RNA, the growing RNA is released from the complex.

In the structure of the EF-Tu and aminoacyl-tRNA (aa-tRNA) complex, domains 2 and 3 of EF-Tu recognize the acceptor helix of the aa-tRNA and the TψC stem and loop of aa-tRNA, respectively [35,36,50]. Some of the residues interacting with the aa-tRNA are also used for the template RNA recognition by the core Qβ eplicase at the elongation stages.

During the RNA elongation stages, no significant structural changes of EF-Tu in the core Qβ replicase are observed. This is consistent with the previous results showing that neither the GTPase activity of EF-Tu nor the TP binding to EF-Tu, which accompanies the structural change of EF-Tu, is required for the RNA polymerization activity of the core Qβ replicase [10,28].

### 4.3. RNA Passages in the Core Qβ Replicase

The sequential crystallographic studies identified the RNA passages in the core Qβ replicase (Figure 5b). The template RNA accesses the catalytic site of the β-subunit through the entrance tunnel, with the 3'-end entering first (Figure 2c). As the RNA chain is elongated, the temporal duplex of the template and growing RNAs proceeds toward EF-Tu in the complex. Subsequently, the duplex is wedged by the *C*-terminal region of the β-subunit, with assistance from EF-Tu, and the single stranded template RNA leaves through the exit tunnel.

The template RNA exit tunnel is formed by the β-subunit and EF-Tu. Once the template RNA is split apart from the growing RNA and the single stranded template RNA is threaded into the exit tunnel, the template RNA would no longer be released from the complex until RNA polymerization is completed. Thus, Qβ virus adopted the unique strategy to recruit a host translational factor for the efficient and complete transcription and replication of its own RNA. Consistent with the structural studies of the elongation stages of RNA polymerization by the core Qβ replicase, the mutations of amino acid residues in EF-Tu that interact with the template RNA reduced the RNA polymerization activity at the elongation stage, rather than the initiation stage [29].

## 5. Termination Stage of RNA Polymerization

### 5.1. Non-Template 3'-A Addition

The 3'-ends of both the positive and negative RNAs are CCA-3', and their 5'-ends are 5'-GG [21,22]. As described above, the 3'-terminal A (3'-A) does not serve as a template nucleoside. Instead, the 3'-penultimate C functions as the first template nucleoside, and is required for the efficient *de novo* initiation of RNA polymerization without using an RNA primer (Figure 1b and Figure 3). Thus, non-template 3'-A addition at the terminal stage of replication in each cycle is required for the efficient replication of Qβ RNA. Indeed, the 3'-terminal nucleoside of the RNAs replicated from Qβ RNA is predominantly adenosine [44,45].

The 3'-A is attached by the intrinsic terminal nucleotidyltransferase activity of Qβ replicase at the terminal stage of RNA polymerization, after template-dependent CC-3' synthesis, without using an RNA template [1,25]. It was previously reported that the non-template 3'-A addition onto Qβ RNA occurs prior to the release of the product from the Qβ replicase, and the Qβ RNA lacking 3'-A cannot accept AMP [1,25]. It was also reported that a particular sequence at the 3'-part of the template RNA is required for the non-template 3'-A addition [26]. The mechanism of non-template 3'-A addition has not been clarified.

### 5.2. Structure of the Termination Stage

**Figure 6:**
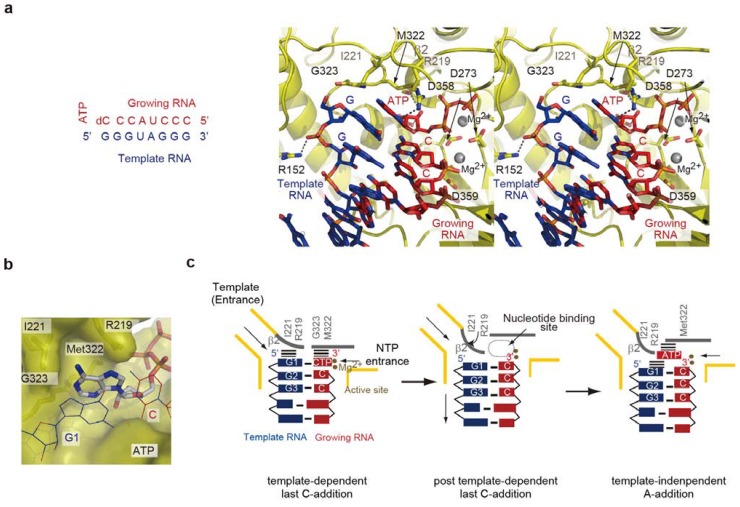
Structures of termination stages. (**a**) Structure of terminal RNA adenylation at the final stage of RNA polymerization by the core Qβ replicase. Only the structure of the catalytic crevasse of the core Qβ replicase is shown. The sequences of the template and growing RNAs are depicted. The template RNA, the growing RNA and ATP are depicted by stick models; (**b**) The size and shape of the pocket are suitable for ATP accommodation; (**c**) Simplified depiction of the structure representing post template-dependent last *C*-addition (**left**) and non-template 3'-A addition (**right**) by the core Qβ replicase. After the last *C*-addition in a template-dependent manner, the double stranded RNA of the template and growing RNAs translocates, and the β2 region changes its conformation and participates in the ATP selection.

The crystal structure of the core Qβ replicase, representing the terminal adenylation of RNA at the final stage of RNA polymerization, was reported [30]. The core Qβ replicase was crystallized with a short template RNA bearing a 5'-G and a growing RNA complementary to the template RNA, except for the 5'-end G, in the presence of 3'-dCTP and ATP. A pre-incubation of the solution allowed us to capture the structure representing the non-template 3'-A addition at the terminal stage of RNA polymerization. In the structure, the geometry of the ATP and the 3'-cytidine incorporated at the 3'-end of the growing RNA, as well as that of the two metals relative to the catalytic carboxylates, suggested that the structure represents the non-template 3'-A addition onto RNA (Figure 6a).

The adenine of the ATP does not specifically interact with the β-subunit, but it is snugly accommodated within the pocket composed of the β-subunit (β2 region) and the 5'-G-C-3' Watson-Crick base pairing, formed at the blunt-ended RNA duplex between the template and growing RNAs. The size and shape of the pocket are suitable for the accommodation of ATP (Figure 6b). The nucleotide binding model showed that CTP and UTP are smaller than the pocket, and GTP is larger than the pocket. These structural features are consistent with the biochemical studies showing that AMP is preferentially incorporated at the 3'-end of RNA at the terminal stage of RNA polymerization. CTP and UTP are not significantly incorporated, and GTP is incorporated less efficiently than ATP [30,45,51].

The structure also suggested that the interaction between the adenine of ATP and the guanine base at the 5'-end of the template RNA stabilizes the ATP accommodation in the pocket. The six-membered ring of the adenine base stacks with the six-membered ring of the guanine base at the 5'-end of the template RNA, through π–π stacking interactions. Biochemical studies using template RNA variants revealed that the adenylation of the 3'-end of the RNA at the terminal stage of RNA polymerization is affected by the sequence of the 5'-part of the template RNA, and the template RNA with the 5'-GG sequence is most competent for non-template 3'-A addition at the terminal stage of RNA polymerization [26,51]. Thus, the continuous stacking interactions between the nucleobases of the 5'-part of the template RNA and the adenine base of ATP, and the strong Watson-Crick base pairing between the 5'-end nucleoside of the template and the 3'-end of the synthesized RNA, contribute to the selection of ATP.

The β2 region, containing Ile221 and Arg219, of the β-subunit undergoes a structural change after the transition from template-dependent RNA synthesis to template-independent 3'-A addition at the terminal stage of RNA polymerization, and also participates in the formation of the ATP binding pocket (Figure 6c). Thus, the template for non-template 3'-A addition is the ribonucleo-protein complex of protein and RNA (Figure 6c). The mechanism of non-template adenylation by the core Qβ replicase is distinct from those of conventional template-independent terminal nucleotidyltransferases, such as CCA-adding enzymes and polyA polymerases [52,53,54,55,56,57,58,59,60].

## 6. Qβ RNA Replication Initiation and Termination

### 6.1. Qβ RNA Replication

Ribosomal protein S1 is an indispensable subunit of Qβ replicase for Qβ RNA replication. In particular, it is required for the synthesis of the negative strand RNA from the positive strand Qβ RNA, while it is dispensable for the synthesis of the positive strand RNA from the negative strand Qβ RNA [1]. In *E. coli*, S1 is the largest protein in ribosomes, and comprises six contiguous oligonucleotide/oligosaccharide-binding (OB)-fold domains (OB1–OB6) [14,61]. The OB-domains are homologous to each other, but the first two *N*-terminal OB-fold domains (OB1 and OB2) are less homologous to the other four. In protein synthesis, S1 is required for the efficient initiation of translation of most natural mRNAs containing a weak or lacking a Shine-Dalgarno (SD) sequence [14,15,16,17,18,19].

Previous studies suggested that S1 binds an internal site on the positive strand Qβ RNA, termed the M-site, about 1400 nucleotides upstream of the 3'-end [1,62]. The sequence of the M-site is required for the efficient initiation of the negative strand RNA synthesis [63,64,65]. Subsequently, long distance interactions were detected between the 3'-part of the positive Qβ RNA and an internal-site downstream of the M-site. These findings suggested that the binding of S1 in Qβ replicase to the M-site of the positive Qβ RNA would recruit the 3'-end of the RNA to the active site of the β-subunit, leading to efficient initiation of negative strand synthesis [66,67]. However, the molecular details of the interactions between S1 and the core Qβ replicase, and the molecular basis of the involvement of the S1 in the RNA polymerization by holo Qβ replicase, have remained elusive.

### 6.2. Structure of the Active Qβ Replicase Containing the S1 Fragment

**Figure 7:**
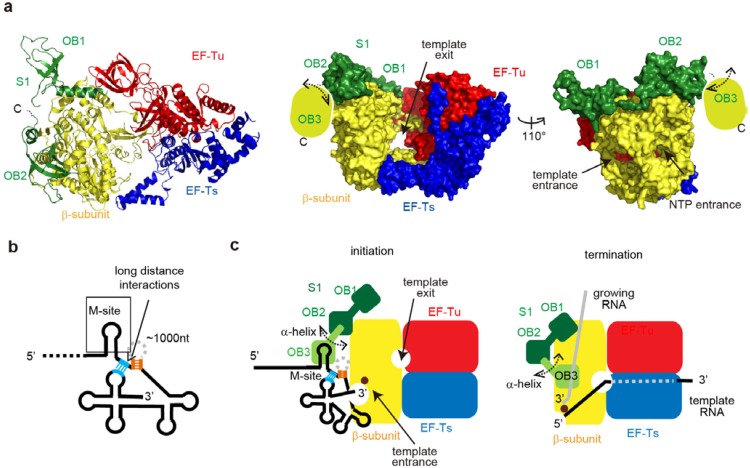
Structure of Qβ replicase containing the *N*-terminal half of S1. (**a**) Overall structure of Qβ replicase containing the *N*-terminal half (oligonucleotide/oligosaccharide-binding (OB)-folds OB1–OB3) of S1. The β-subunit, EF-Tu, EF-Ts, and OB1-2 are colored yellow, red, blue, and green, respectively. OB3 is mobile, and its model could not be built in the structure; (**b**) Schematic two-dimensional view of the 3'-half of Qβ RNA. The long-distance interactions in the RNA are depicted by dotted lines, and the regions used for interactions in the Qβ RNA are colored orange and sky blue; (**c**) Simplified schematic depictions of Qβ RNA replication initiation (**left**) and termination (**right**). The OB3 domain is mobile and capable of pivotal rotation, using the α helix connecting OB2 and OB3 as a swing arm.

Recently, the crystal structure of Qβ replicase containing the *N*-terminal three OB-fold domains (OB1, OB2 and OB3) of S1 was analyzed [31]. The complex is capable of synthesizing negative strand RNA from the positive strand Qβ RNA, and the RNA synthesis depends on the M-site in the positive Qβ RNA, similar to the holo Qβ replicase [31]. The stoichiometry of the β-subunit, EF-Tu, EF-Ts, and S1 protein variant (OB1–3) in the asymmetric unit is 1:1:1:1. S1 only binds to the β-subunit of the core Qβ replicase, via its *N*-terminal OB1 and OB2 domains (Figure 7a) [68]. The OB3 domain itself does not interact with the β-subunit, and it is exposed to the solvent and relatively mobile, but is spatially anchored near the surface of the β-subunit.

Biochemical studies revealed that the *N*-terminal half of S1 can bind a short RNA fragment derived from the M-site region in the positive strand Qβ RNA. The third *N*-terminal OB-fold (OB-3) is the primary domain for the binding. The *N*-terminal two OB-folds (OB1 and OB2) lack the RNA binding residues, and would lack ability to bind the RNA fragment significantly. The OB3 would cooperatively function together with OB1 and OB2. *In vitro* negative strand RNA synthesis from positive strand Qβ RNA indicated that the RNA-binding ability of the OB3 domain of S1 correlates with the replication initiation efficiency. Thus, the OB3 domain of S1, which is specially anchored near the surface of the β-subunit, specifically recognizes the distinct sequence in the internal region of Qβ RNA together with OB1 and OB2 cooperatively.

Previous studies showed that long-distance interactions, between the 3'-terminal region of Qβ RNA and an internal-site just downstream of the M-site, are required for the efficient initiation of Qβ RNA replication (Figure 7b) [66,67]. Thus, the specific recognition of the internal site of Qβ RNA by the OB-3 domain itself or together with the two *N*-terminal OB-folds (OB1 and OB2) allows the 3'-region of Qβ RNA to be located in the proximity of the active site of the β-subunit (Figure 7a,c). This would enhance the efficient and specific initiation of Qβ RNA replication.

Recent biochemical studies also suggested that the *N*-terminal half of S1, containing OB1-3, is sufficient for the efficient release of the product RNA from the template RNA in a single stranded form at the terminal stage [69]. The OB3 domain in the *N*-terminal half of S1 is mobile and would be capable of pivotal rotation, using the α helix between OB2 and OB3 as a swing arm. Therefore, the OB3 domain in S1 also acts as a termination factor, by binding to the growing RNA and preventing the template and growing RNAs from forming double stranded RNA (Figure 7c). At present, it is not experimentally demonstrated whether the RNA-binding ability of the OB3 domain of S1 correlates with the efficient release of the product RNA from the template RNA in a single stranded. Further study would clarify this point.

Taken together, S1 in Qβ replicase can act as a replication initiation factor as well as a termination factor. In ribosomes, S1 is anchored onto the 30S ribosome via its two *N*-terminal OB-fold domains [14,20], and the *N*-terminal half of S1 is required and sufficient for the initiation of mRNA translation [20]. Thus, the functional domains of S1 in Qβ replicase that are required for the initiation of Qβ RNA replication are also necessary in the ribosome for translation initiation.

## 7. Conclusions

Replication and transcription of viral RNA in host cells rely on the host-derived factors, although the catalytic subunits, RdRps, for RNA synthesis are derived from the viral RNA. In some RNA viruses from eubacteria, plant and animal origins, the viral RdRps form a complex with the host-derived translational factors [70]. The RdRps from plant and animal viruses form replicative complexes with host translation factors, such as eIF3 (bromo mosaic virus and tobacco mosaic virus), eEF1α (polio virus), and eEF1 α β γ (vascular stomatitis virus) [71,72,73,74]. The RdRps from eubacteria viruses, such as Qβ virus, form a complex with EF-Tu, EF-Ts and ribosomal protein S1 [1].

In this review, we have described recent reports leading toward an understanding of RNA polymerization by Qβ replicase. In particular, the alternative functions of the host translation factors in Qβ replicase, beyond their functions in protein synthesis, were presented based on the recent crystallographic analysis of Qβ replicase as well as biochemical studies.

Structural studies of the core Qβ replicase representing RNA polymerization revealed that domains 2 and 3 of EF-Tu are important for the separation of the double strand RNA between the template and growing RNAs, for efficient and processive RNA polymerization (Figure 4). Together with the β-subunit, these domains compose the exit tunnel for the single stranded template RNA separated from the growing RNA. Domains 2 and 3 of EF-Tu are utilized for binding the aminoacyl-tRNA, in the elongation cycle of protein synthesis. A structural study of the core Qβ replicase with the *N*-terminal half of S1 also revealed that S1 is anchored to the β-subunit of the core Qβ replicase via its two *N*-terminal OB-folds, while the third, mobile OB-fold is important for the recognition of Qβ RNA, the efficient initiation of Qβ RNA, and termination (Figure 7c). S1 interacts with the ribosome via the two *N*-terminal two OB-folds, and the third OB-fold is required for translation initiation. 

The translational elongation factors EF-Tu and EF-Ts and the ribosomal protein S1 are all essential for protein synthesis in *E. coli*. Thus, *E. coli* would not be tolerant to mutations in the functional domains of these translation factors. Qβ virus might have evolved to employ a smart strategy to recruit these essential host factors and utilize their functionally important domains for its propagation in the host cells.

Alternatively, these translational factors might have been original cofactors required for RNA replication, and the modern translational apparatus might have borrowed these replication factors for protein synthesis [29,75]. Translational elongation factor, EF-Tu, in Qβ replicase functions as an RNA replication factor for the efficient and complete replication and transcription of the viral RNA. Especially, the RNA-binding domains of EF-Tu, domains 2 and 3, interact with the template RNA during the elongation process of replication and transcription of the viral RNA (Figure 5), and RNA binding domains (OB-folds) of S1 interact with template and growing RNAs at initiation and termination stages of replication, respectively (Figure 7). Thus, the RNA binding domains, such as OB fold proteins, of translation factors of EF-Tu and S1 might be a kind of “molecular fossil” in the modern living systems, and might have been the ancestors of modern translational factors. Ancient RNA binding proteins, required for RNA replication systems, might have acquired additional functional domains, and functions in the modern translational apparatus in more sophisticated and specified manners.
